# Survival benefit of surgery to patients with esophageal squamous cell carcinoma

**DOI:** 10.1038/srep46139

**Published:** 2017-04-06

**Authors:** Miao-Fen Chen, Ping-Tsung Chen, Ming- Shian Lu, Chuan-Pin Lee, Wen-Cheng Chen

**Affiliations:** 1 Department of Radiation Oncology, Chang Gung Memorial Hospital at Chiayi, Taiwan; 2 Chang Gung University College of Medicine, Taiwan; 3 Hematology and Oncology, Chang Gung Memorial Hospital at Chiayi, Taiwan; 4 Thoracic & Cardiovascular Surgery, Chang Gung Memorial Hospital at Chiayi, Taiwan; 5 Center of Excellence for Chang Gung Research Datalink, Chang Gung Memorial Hospital, Chiayi, Taiwan

## Abstract

To assess if surgery provided survival benefit to patients with esophageal squamous cell carcinoma (SCC), we performed a retrospective review of 1230 patients who were newly diagnosed with stage T2-T4 esophageal SCC from 2007 to 2014 in our hospital. There were greater than 70% of patients with age under 65 years, and more than 85% were stage T3-T4 at the time of diagnosis. The median survival time was 1.06 year (95% CI 0.99–1.1 yrs). Survival analyses showed that survival time was significantly associated with age, T stage, clinical lymph node involvement and treatment modality (surgery *versus* definite chemoradiotherapy). Surgery still possessed a powerful impact on overall survival by multivariable analysis. Death risk of patients treated with curative surgery was significantly lower than those with definite chemoradiotherapy. Furthermore, for patients of stage T3N(+) and T4, surgery combined with (neo-)adjuvant treatment were significantly associated with higher survival rate than surgery alone or definite chemoradiotherapy. In conclusion, the patients who undergo surgery were significantly associated longer survival, therefore, curative resection should be considered for esophageal cancer patients who are medically fit for surgery. Moreover, combined with (neo-)adjuvant treatment is recommended for surgically resectable stage T3-T4 esophageal SCC.

Esophageal carcinoma is one of the most common cancers, and considered a serious malignancy with respect to prognosis and mortality rate^1^. Despite many advances in diagnosis and treatment, esophageal cancer still is an aggressive disease, characterized by a high degree of locoregional and distant recurrence and poor overall survival^2^,^3^. Surgery is a major component of treatment for resectable esophageal cancer, especially for adenocarcinoma. Surgery has been regarded as mainstay of cure for esophageal cancer in the past although distant control and complete resection rate remain issues with surgery^4^,^5^. The postoperative mortality^6^,^7^ and higher rate of relapse with esophagectomy have prompted investigation of multidisciplinary management, such as concomitant chemoradiotherapy (CCRT) with or without surgery^8^,^9^,^10^. However, the most appropriate treatment modality for esophageal cancer is still controversial. Within the past decade, several studies investigating the curative potential of CCRT have challenged the idea that surgery is an indispensable part of curative therapy^11^,^12^. Factors involved in the treatment decision include baseline clinical stage, location of the primary, and histology. Our previous data indicated that age, sex, and curative treatment were significant predictors of survival in patients with esophageal cancers lacking cancer stage and histology information^13^. Esophageal cancer exists as two distinct histological types: squamous cell carcinoma (SCC) and adenocarcinoma. There are marked differences between these carcinomas in terms of incidence, natural history and treatment outcomes^14^,^15^,^16^. Adenocarcinoma now is the leading cause of esophageal cancer in the United States, representing 80% of cases^17^. The question whether operable SCC of the esophageal cancer should be treated by radiotherapy or resection has been posed already three decades ago. Greater than 90% patients with esophageal cancer in our hospital have SCC. Therefore, we focus the role of surgery in the treatment of esophageal SCC compared with CCRT in the present study. It includes whether operable esophageal SCC should be treated by radiotherapy, resection or combined treatment, and if surgery provide a survival benefit in multidisciplinary treatment compared with definite CCRT for locally advanced esophageal SCC.

## Results

### Overall survival of patients with esophageal SCC

**Figure 1:**
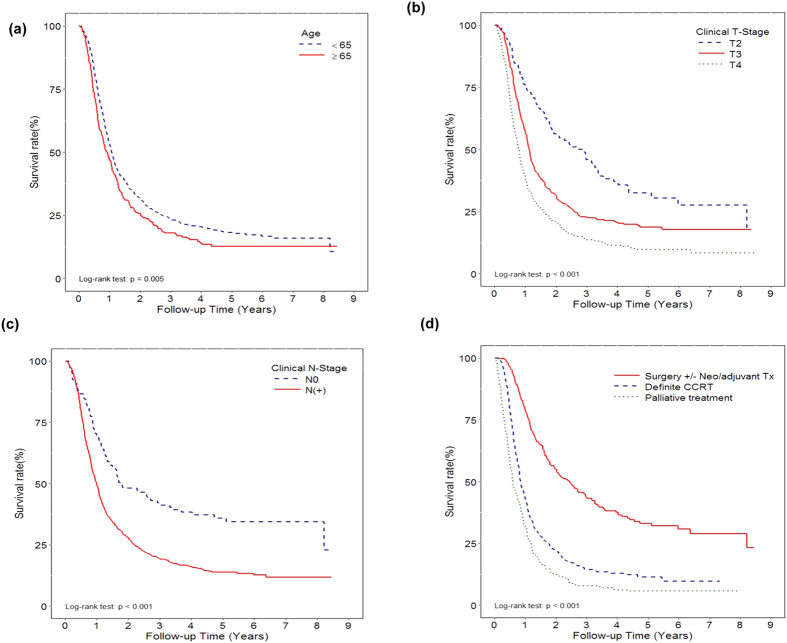
Overall survival in patients with T2-T4 esophageal SCC Kaplan-Meier survival curves of the total 1230 patients with clinical stage T2-T4 and histology confirmed with SCC; and the survival differences according to age (**a**), clinical T stage (**b**), and clinical lymph node involvement (**c**). Additionally, Kaplan-Meier survival curves according to treatment modality (Surgery+/−Neo/adjuvant Tx versus definite CCRT versus palliative treatment) (**d**).

From 2007 to 2014, there were 1309 new cases diagnosed with esophageal cancer and with clinical stage T2-T4 and in our hospital. Among these patients, more than 90% was diagnosed with histologically confirmed SCC of esophagus. For clinical stage T2-T4 esophageal cancer, the median survival times were 1.06 year (95% CI 0.99-1.1 yrs) for patients with SCC, and 1.58 year (95% CI 0.85-3.21 yrs) for adenocarcinoma. To focus the role of surgery in the treatment of esophageal SCC, we conducted all further analysis on the basis of 1230 patients with T2-T4 esophageal SCC. The clinical characteristics are listed in Table 1. In our hospital, approximately 15 times as many males as females (1152 males and 78 females) had esophageal SCC diagnosed, and 30% patients received surgery as the major treatment modality for esophageal SCC. By survival analysis for patients with T2-T4 esophageal SCC (Fig. 1a), increasing age was associated with lower survival rates. In addition, as shown in Fig. 1b–c, clinical T stage and clinical lymph node (LN) involvement were significant predictor for prognosis. The 3-year survival rate was 45.9%; 22.9% and 13.7% for clinical T2, T3 and T4, respectively. The median survival time was 1.8 years (95% CI 1.45-2.96 yrs) in patients with clinical N0 and 1 year (95% CI 0.93-1.07 yrs) in those with clinical LN involvement.

### Survival Rate of T2-T4 esophageal SCC by Treatment Modality

**Figure 2:**
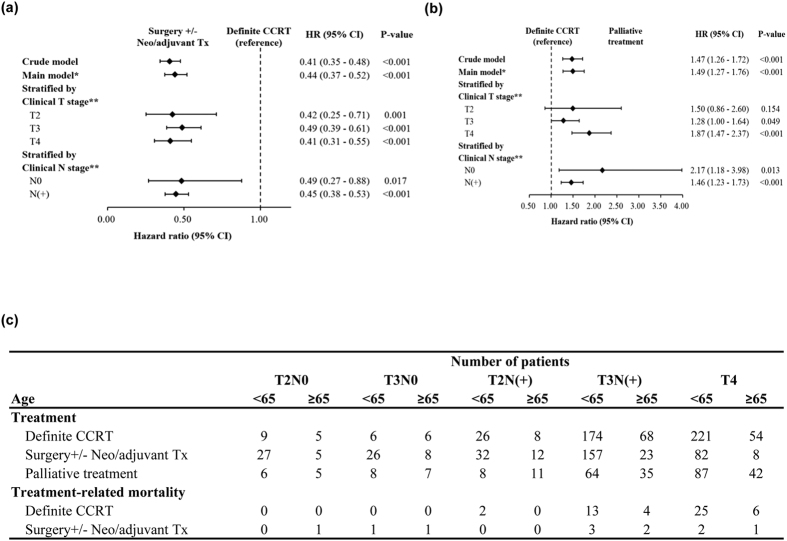
Treatment modality associated with prognosis of patients in multivariate model for patients with T2-T4 esophageal SCC Multivariable Cox regression analysis of based on different co-variables in patients with T2-T4 (**a**) Surgery+/−Neo/adjuvant Tx versus definite CCRT; and (**b**) definite CCRT versus palliative treatment. (**c**) The details of treatment modality and treatment-related modality for each stage. (*Hazard ratios estimated in the main model were adjusted for age, gender and clinical stage; **Hazard ratios were estimated by fitting the main model within the stratified subgroup).

We further analyzed survival rate by treatment modality for patients with T2-T4 esophageal SCC. The overall 1- and 3-year survival rates after diagnosis were 52, and 22%, respectively. As shown in Fig. 1d, patients received surgery with or without (neo-) adjuvant treatment had significantly longer survival than patients treated with definite radiotherapy and chemotherapy. The median survival time was 2.46 years (95% CI 1.98-2.96 yrs) in patients treated with surgery, 0.85 years (95% CI 0.82-0.95 yrs) in those with definite CCRT, and 0.61 years (95% CI 0.54-0.76 yrs) in those with supportive or palliative treatment. Further multivariable Cox regression analysis of based on different co-variables showed that surgery with or without (neo-) adjuvant treatment could reduce more than 50% risk of death in T2-T4 stage, compared to those treated with definite CCRT (P < 0.001) (Fig. 2a). Figure 2b demonstrated that definite CCRT significantly improved survival compared to those only received palliative treatment. In addition, the details of treatment modality and treatment-related modality for each stage were shown in Fig. 2c. The data revealed that the treatment-related mortality (death occurred during or within 30 days after the completion treatment) rates in patients treated with curative intent surgery or CCRT. By the data, we found that surgery with or without (neo-)adjuvant treatment had no significant impact on treatment-mortality rate compared with that in CCRT group.

### Treatment outcome of esophageal SCC by clinical stage

**Figure 3:**
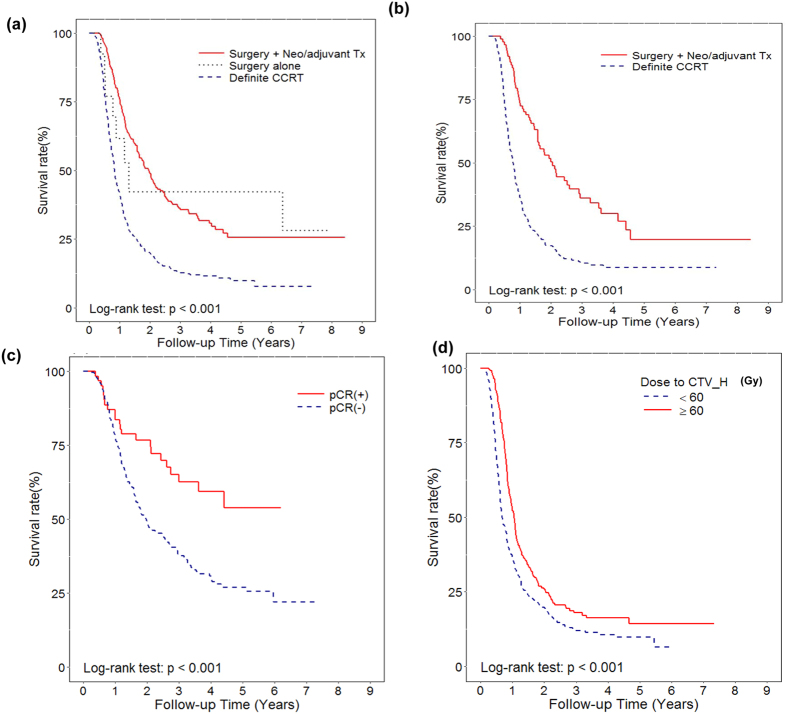
Overall survivals in patients with esophageal SCC in subgroups of clinical stage and treatmentThe survival differences according to curative treatment modality in patients with T3N(+) and T4 (Surgery alone versus Surgery+Neo/adjuvant Tx versus definite CCRT) (**a**); and patients with stage T4N0-N(+) (Surgery+Neo/adjuvant Tx versus definite CCRT) (**b**). Additionally, Kaplan-Meier survival curves for patients with stage T2-T4 in the surgery group according to achieving pathologic complete response or not (**c**), and in the CCRT group according to the dose of radiotherapy (**d**).

For patients with clinical stage T2-T3 esophageal SCC, as shown in Table 2, clinical T3 stage, clinical LN involvement, older ages, and no surgical resection were significantly associated with shorter survival time in univariate survival analysis. Further multivariable Cox regression analysis of based on different co-variables showed that surgery with or without (neo-) adjuvant treatment could reduce more than 50% risk of death in T2-T3 stage, compared to those treated with definite CCRT (Fig. 2a and Table 3). Furthermore, for the subgroup of patients with T3N(+) and T4, surgery combined with (neo-)adjuvant treatment obviously prolonged the survival rate compared to surgery alone or definite CCRT (Fig. 3a) (surgery alone versus definite CCRT, *p* = 0.179; surgery+ (neo-)adjuvant treatment versus definite CCRT, *p* < 0.001). For stage T4 esophageal SCC, there were only 90 patents (18%) received curative intent surgery. As shown in Fig. 3b, surgery combined with (neo-) adjuvant treatment provided the better overall survival rate than definite CCRT, with the median survival was 2.05 years (95% CI 1.57-2.90 yrs) and 0.78 years (95% CI 0.68-0.84 yrs), respectively. As shown in Fig. 2a and Table 4, multivariable survival analysis, surgery combined with (neo-)adjuvant treatment decreased 58% death risk compared to those treated with definite CCRT. In surgery group of patient with stage T2-T4, as shown in Fig. 3c, achieving pathologic complete response (pCR) after neoadjuvant treatment is a significant predictor for longer survival. For stage T2-T3, the 3-yr survival rate was 63.6% for patients with pCR, and 41.6% for patients without pCR (p = 0.013), respectively. For T4 esophageal SCC, the median survival is 4.41 years in patients received surgery with pathologic complete response, and 1.58 years in patients without pCR after neoadjuvant treatment. In addition, patients who treated with definite CCRT had significantly improved survival compared to those only received palliative treatment. In the group of definite CCRT, the radiation dose is the significant predictors for better overall survival (Fig. 3d).

## Discussion

This was a retrospective study of the factors influencing the survival of patients with esophageal cancer, using the data of cancer registry and death registration in our hospital from 2007 to 2014. We previously showed that the survival of esophageal cancer patients who underwent surgery improved significantly compared with definite radiotherapy^13^. However, the limitations were the absence of the data about tumor stages, histologic tumor type and the details of treatment modalities. Therefore, an advantage of our analysis in the present study is that the results are based on a relative large population of esophageal cancer patients with information regarding tumor histology, staging and primary treatment detail.

In the western world, esophageal cancer has been considered a disease of the older population with a peak incidence between the sixth and seventh decades of life, and adenocarcinoma predominates^17^,^18^,^19^. According to the data of our hospital, the age of peak incidence of esophageal cancer was 50-64 years. Unsurprisingly, increasing age (>65 y/o) was associated with a marked decrease in overall survival rates similar to other studies^20^,^21^. The prevalence of esophageal cancer exhibited a marked sex difference, and it was a significant predictor for prognosis for T2-T3but not for T4 esophageal SCC.

For esophageal cancer, there are marked differences between adenocarcinoma and SCC in terms of natural history and treatment outcomes^14^,^15^,^16^. Although surgery is a major locoregional treatment^5^,^22^,^23^, the curative potential of CCRT reported in several studies have challenged the role of surgery in esophageal cancer^11^,^12^. The most appropriate treatment modality for esophageal SCC is still controversial. Greater than 90% patients with esophageal cancer in our hospital have SCC. Therefore, we focus on if surgery provides a survival benefit in esophageal SCC in the present study. Multivariate analysis of based on different co-variables showed that surgery with or without (neo-) adjuvant treatment could reduce more than 50% risk of death in T2-T4 stage, compared to those treated with definite CCRT. Furthermore, compared to patients without curative treatment, definite CCRT significantly increased the overall survival rate. The appropriate radiation dose for definite CCRT is controversial. Although 50.4 Gy remains the Radiation therapy Oncology Group (RTOG) standard dose for radiation with delivered concurrently with chemotherapy^24^,^25^, RT dose up to at least 60 Gy was used in randomized trials comparing definitive CCRT vs surgery^11^,^12^,^26^. Our survival analysis revealed that definite CCRT had lower survival rate than that underwent surgical resection, but the higher doses of radiation (>=60Gy) is significant benefit to survival. Therefore, by our database, surgery provided a survival benefit compared to definite CCRT, and radiotherapy dose should be higher than 60Gy for patients treated with definite CCRT.

It has been reported that the 5-year survival rate post-esophagectomy was 52% for patients with no residual tumor, but decreased to only 14% if residual tumors were present^27^. To increase the possibility of complete resection, more than 85% of patients with T2-3N (+) and T4 who underwent surgical resection received (neo-) adjuvant treatment in our hospital. For esophageal adenocarcinoma, it has been reported that (neo-) adjuvant radiotherapy are of significant survival benefit to T3N0M0 stage, but not significantly good to T2N0M0^28^. By the database in our hospital, (neo-) adjuvant treatment had significant survival benefit to stage T3N(+) and T4, but not to stage T2N(+). Furthermore, in surgery group of stage T2-T4, univariate survival analyses showed that survival time was associated with clinical T stage, LN involvement and pathologic complete response. The presence of pathologic complete response (pCR) has been shown to be a significant prognostic factor for local and distant recurrence in esophageal cancer patients receiving trimodality therapy^29^,^30^,^31^. The pCR rate of 18% in our study was low relative to that reported in other studies ranged from 20% to 40%^32^,^33^,^34^. The potential reasons included the difference in chemotherapy agents, radiotherapy doses received, as well as the quality and expertise of the pathologists who analyzed the surgical specimen. Treatment-related mortality is an important issue to guide if multidisciplinary therapy is benefit for survival^35^. In patients with stage T2-4, (neo-)adjuvant treatment didn’t increase the treatment-related mortality in surgery group of our hospital. Therefore, improvement in surgical techniques and perioperative risk evaluation might explain why surgery significantly increased overall survival rate of esophageal cancer patients demonstrated by multivariable analysis, at least in part.

The reported incidence of stage T4 is 12–34% among thoracic esophageal cancer^36^,^37^ with a false positive rate of approximately 40%^38^,^39^. For T4 esophageal SCC, it is still unclear if the addition of surgery provided survival benefit in patients with compared with definite CCRT. Previous studies reported the effectiveness of definite CCRT in advanced esophageal cancer including T4 tumors^40^,^41^. Although only 18% of T4 patients in our hospital received surgical resection, survival analysis showed that surgery with (neo-) adjuvant treatment offers a favorable overall survival compared with definite CCRT by uni- and multivariable analysis. With respect to superior local control and higher false positive rate of clinical T4, it is an important issue about what population of patients with T4 tumors would achieve survival benefit by undergoing resection after neoadjuvant CCRT^40^. Randomized controlled trials involving large population samples are needed to define the standard treatment for T4 esophageal cancer.

The limitations to our study are related to the inherent nature of investigating a hospital-based registry. We are unable to ascertain the reason for the delay in initiation of curative treatment or the choice of palliative treatment. Furthermore, we could not account for potential unmeasured selection biases regarding performance status, comorbidity, access to health care, or other patient-related factors.

## Conclusions

Esophageal cancer is a particularly devastating form of cancer with a relatively low survival rate. By outcome analysis, we demonstrated that treatment with surgery is significantly linked with better survival rate in patients with esophageal SCC. Therefore, curative resection should be considered for esophageal cancer patients who are medically fit for surgery.

## Materials and Methods

### Data source

The data source comes from our hospital Cancer Registry and death registration (CGRD) in this study. CGRD is a high quality cancer registry and provides sufficient information regarding individual demographics, stage of disease, tumor histology, and primary treatment details.

### Study population and study design

**Figure 4:**
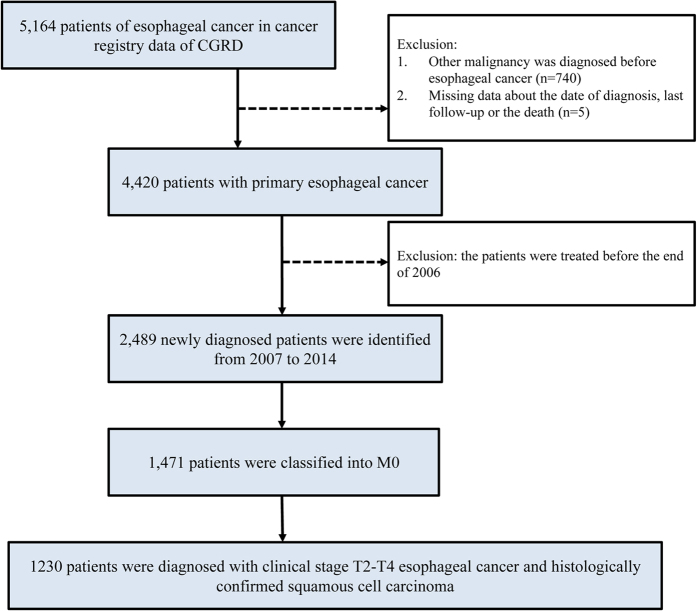
Study flow chart We enrolled the patients with clinical stage T2-T4N0-N(+) esophageal SCC into our present study. For clinical stage, 6th American Joint Committee on Cancer staging were used in 2007–2009 or 7th stage in 2010–2014.

This study adhered to strict confidentiality guidelines, in accordance with regulations regarding personal electronic data protection, and was approved by the Institutional Review Board of Chang Gung Memorial hospital. From the database, we included all subjects who were newly diagnosed with esophageal cancer between January 1, 2007 and December 31, 2014, and all medical records of the esophageal cancer cohort were extracted and analyzed. The patients with other cancers diagnosis before the first day of esophageal cancer diagnosis in the records of CGRD was excluded from this study. All enrolled study subjects were followed-up until death or the end of 2015. Our study flow chart was depicted in Fig. 4. From 2007 to 2014, CGRD database provided a total 2489 cases in whom esophageal cancer was diagnosed as the first malignancy, and complete data were available for analysis. We further excluded 1018 patients with stage M1 and 162 patients with clinical stage Tis-T1 at diagnosis from this study. To focus the prognosis of esophageal SCC, a total of 1230 patients had a diagnosis of histologically confirmed SCC of esophagus with clinical stage T2-T4 enrolled into our study. The curative treatment for T2-T4 esophageal cancer was according to the guidelines proposed by oncology team at our hospital. Surgery is considered for all physiologically fit patients with localized, resectable, esophageal cancer. If surgery was contraindicated or the patients refused it, they received definite CCRT with radiotherapy dose for 40–66Gy. In this study, we included patient demographic (age, gender), disease characteristics (tumor location, clinical T-stage and N-stage), and treatment characteristics.

### Statistical analysis

We used the Kaplan-Meier method to calculate survival curves and the log-rank test to compare the two groups for difference in survival curves. Finally, Cox proportional hazards models were used to compute the hazard ratios (HRs) accompanying 95% confidence intervals (CIs) after adjustment for esophageal cancer treatment and clinical characteristics. All of these analyses were conducted using SAS statistical software (version 9.2; SAS Institute, Cary, NC, USA).

## Additional Information

**How to cite this article**: Chen, M.-F. *et al*. Survival benefit of surgery to patients with esophageal squamous cell carcinoma. *Sci. Rep.* **7**, 46139; doi: 10.1038/srep46139 (2017).

**Publisher's note:** Springer Nature remains neutral with regard to jurisdictional claims in published maps and institutional affiliations.

## Figures and Tables

**Table 1:** Characteristics of patients with T2-T4 esophageal SCC.

	Patients Treatment		
	Definite CCRT	Surgery+/− Neo/adjuvant Tx	Palliative treatment
Number (%)	577 (100%)	380 (100%)	273 (100%)
Age			
<50y/o	168 (29.1%)	119 (31.3%)	62 (22.7%)
50–64y/o	268 (46.4%)	205 (53.9%)	111 (40.7%)
>64y/o	141 (24.4%)	56 (14.7%)	100 (36.6%)
Gender			
Female	35 (6.1%)	16 (4.2%)	27 (9.9%)
Male	542 (93.9%)	364 (95.8%)	246 (90.1%)
Clinical T stage			
T2	48 (8.3%)	76 (20.0%)	30 (11.0%)
T3	254 (44.0%)	214 (56.3%)	114 (41.8%)
T4	275 (47.7%)	90 (23.7%)	129 (47.3%)
Clinical N stage			
N0	31 (5.4%)	72 (18.9%)	41 (15.0%)
N1	205 (35.5%)	150 (39.5%)	169 (61.9%)
N2	217 (37.6%)	114 (30.0%)	43 (15.8%)
N3	124 (21.5%)	44 (11.6%)	20 (7.3%)

Abbreviations: Neo/adjuvant Tx = neoadjuvant or adjuvant radiotherapy+/− chemotherapy; CCRT = concomitant chemoradiotherapy.

**Table 2:** Adjusted hazard ratio of determine factors associated with prognosis of patients in univariate model for patients with T2-T3 esophageal SCC.

Variable	HR	95% CI	P value
Age			
<65	Ref		
>=65	1.35	1.12–1.63	0.002
Gender			
Female	Ref		
Male	1.48	1.00–2.18	0.048
Clinical T stage			
T2	Ref		
T3	1.80	1.43–2.27	<0.001
Clinical N stage			
N0	Ref		
N(+)	1.85	1.43–2.40	<0.001
Treatment			
Definite CCRT	Ref		
Surgery+/−Neo/adjuvant Tx	0.44	0.36–0.54	<0.001
Palliative treatment	1.31	1.05–1.62	0.015

**Table 3:** Adjusted hazard ratio of determine factors associated with prognosis of patients in multivariate model for patients with T2-T3 esophageal cancer.

Variable	HR*	95% CI	P value
Age			
<65	Ref		
≥65	1.23	1.01–1.50	0.037
Gender			
Female	Ref		
Male	1.64	1.10–2.44	0.016
Clinical T stage			
T2	Ref		
T3	1.57	1.23–1.99	<0.001
Clinical N stage			
N0	Ref		
N(+)	1.30	0.98–1.71	0.065
Treatment			
Definite CCRT	Ref		
Surgery+/−Neo/adjuvant Tx	0.48	0.39–0.59	<0.001
Palliative treatment	1.34	1.08–1.67	0.008

*HRs were estimated for multivariable Cox model with the variables listed in the table.

**Table 4:** Adjusted hazard ratio of determine factors associated with prognosis of patients in multivariate model for patients with T4 esophageal cancer.

Variable	HR*	95% CI	P value
Age			
<65	Ref		
≥65	0.90	0.71–1.14	0.385
Gender			
Female	Ref		
Male	0.91	0.62–1.33	0.619
Clinical N stage			
N0	Ref		
N(+)	1.51	0.95–2.39	0.084
Treatment			
Definite CCRT	Ref		
Surgery+/−Neo/adjuvant Tx	0.42	0.31–0.56	<0.001
Palliative treatment	1.92	1.53–2.42	<0.001

*HRs were estimated for multivariable Cox model with the variables listed in the table.
